# Genetic Diversity and Identification of Chinese-Grown Pecan Using ISSR and SSR Markers

**DOI:** 10.3390/molecules161210078

**Published:** 2011-12-06

**Authors:** Xiao-Dong Jia, Tao Wang, Min Zhai, Yong-Rong Li, Zhong-Ren Guo

**Affiliations:** 1 Institute of Botany, Jiangsu Province and Chinese Academy of Sciences, Nanjing Botanical Garden Mem. Sun Yat-Sen, Nanjing 210014, China; Email: jiaxiaodong@cnbg.net (X.-D.J.); wxtao@sina.cn (T.W.); 2 Nanjing LvZhou pecan Co., Ltd, Nanjing 210007, China; Email: zhaimin13849058867@126.com (M.Z.); ll194288@yahoo.com.cn (Y.-R.L.)

**Keywords:** genetic diversity, pecan, ISSR, SSR

## Abstract

Pecan is an important horticultural nut crop originally from North America and now widely cultivated in China for its high ecological, ornamental and economic value. Currently, there are over one hundred cultivars grown in China, including introduced American cultivars and Chinese seedling breeding cultivars. Molecular markers were used to assess the genetic diversity of these cultivars and to identify the pedigrees of fine pecan plants with good characteristics and no cultivar-related data. A total of 77 samples grown in China were studied, including 14 introduced cultivars, 12 domestic seedling breeding cultivars, and 49 fine pecan plants with no cultivar data, together with *Carya cathayensis* and *Juglans nigra*. A total of 77 ISSR and 19 SSR primers were prescreened; 10 ISSR and eight SSR primers were selected, yielding a total of 94 amplified bands (100% polymorphic) in the range of 140–1,950 bp for the ISSR and 70 amplified bands (100% polymorphic) in the range of 50–350 bp for SSR markers. Genetic diversity analyses indicated Chinese-grown pecan cultivars and fine plants had significant diversity at the DNA level. The dengrograms constructed with ISSR, SSR or combined data were very similar, but showed very weak grouping association with morphological characters. However, the progeny were always grouped with the parents. The great diversity found among the Chinese cultivars and the interesting germplasm of the fine pecan plants analyzed in this study are very useful for increasing the diversity of the pecan gene pool. All 77 accessions in this study could be separated based on the ISSR and SSR fingerprints produced by one or more primers. The results of our study also showed that ISSR and SSR techniques were both suitable for genetic diversity analyses and the identification of pecan resources.

## 1. Introduction

Pecan (*Carya illinoensis*) is a North American deciduous tree that belongs to the *Juglandaceae* family, which includes hickories, walnuts and the *Carya* genus, which comprises approximately 25 species of large trees. In North America, the pecan tree is native to the Mississippi River Valley and its tributaries, and its natural distribution extends approximately from latitude 26° to 42° and longitude 84° to 103°, primarily along rivers. The range of cultivation extends from North Carolina to Southwest California [1]. Pecan is one of the most important horticultural nut crops in the World and been introduced into China in the 1900s. Now there are over one hundred cultivars in China, including cultivars introduced from America and domestic seedling breeding cultivars [2]. Domestic cultivars always exhibit very suitable local climate preferences and good characteristics, but lack genetic data. We also wanted to use genetic fingerprinting to clarify some confusion related to the cultivars, which may have been caused by the old-age introduction, incomplete or erroneous records or other reasons.

In previous works, the genetic diversity of pecan cultivars was assessed in different ways. Several isozyme systems had been developed and used for studying the genetic diversity of pecan populations [3,4,5,6,7,8] and identifying pecan cultivars [9]. However, the relatively small number of isozyme markers available reduces their utility in fingerprinting and assessing the genetic relationships among cultivars. Molecular marker techniques have since been implemented for pecan genetic diversity analysis. For example, Vendrame used amplified fragment length polymorphisms (AFLPs) for the molecular evaluation of pecan trees regenerated from somatic embryogenic cultures [10], and Conner and Wood used randomly amplified polymorphic DNAs (RAPDs) to identify pecan cultivars and estimate their genetic relatedness [11]. Grauke *et al.* had developed and evaluated simple sequence repeat (SSR) markers for the genetic studies of pecan [12]; 19 SSR primers have been evaluated, with 11 revealing polymorphism. Beedanagari *et al.* have constructed the first linkage map of pecan, using RAPD and AFLP markers [13]. In China, Zhang *et al.* used RAPD to analyze the population genetic diversity of 30 introduced American pecan cultivars, although Chinese cultivars were not investigated [14,15]. From previous studies of pecan, it is clear that these molecular marker techniques were only focused on the American pecan cultivars, whereas the Chinese-grown pecans, which are very suited to the local climate and possess many great characters, were not investigated.

Both inter-simple sequence repeat (ISSR) and SSR markers based on the polymerase chain reaction (PCR) are commonly used for measuring genetic diversity [16,17] and for cultivar identification [18,19]. ISSR analyses are easy to perform, are inexpensive, and do not require prior genetic information [20]. SSR markers are also very useful markers in genetic diversity estimates, genetic mapping and DNA fingerprinting due to their high level of reproducibility and transportability across laboratories [21]. The combination of these two techniques has been used effectively for the identification and assessment of germplasm in plants, including barley and yardlong bean [22,23].

An understanding of the genetic relationships of the pecan germplasm is difficult because of the inherent difficulties of working with a large perennial tree, but it is vital for continuing any breeding program that endeavors to increase the genetic diversity of new cultivars. In this study, we fingerprinted 77 pecan samples grown in China, including 14 introduced cultivars, 12 domestic seedling breeding cultivars, 49 fine pecan plants with no cultivar data, and *Carya cathayensis* Sarg. and *Juglans nigra* Linn., by screening 77 ISSR and 19 SSR markers. Using 10 ISSR and eight SSR markers, we addressed three objectives: (1) to detect the genetic diversity and genetic variation among the introduced American cultivars, Chinese cultivars and fine pecan plants; (2) to identify (or fingerprint) different cultivars and fine plants; and (3) to discuss the genetic relatedness between the ISSR and SSR markers. Our long-term goal is to select suitable varieties for cultivation in China. These results should provide useful data for further breeding programs.

## 2. Results and Discussion

The following 77 plant samples were used in this study: 14 cultivars introduced from America, 12 Chinese seedling breeding cultivars, 49 fine pecan plants with no cultivar data, *Carya cathayensis* and *Juglans Nigra*. A list of the samples investigated is given in Table 1.

**Table 1:** List and description of the 77 accessions used in this study.

No.	Name	Type ^a^	Origin ^b^	Pollination	Harvest Season	Scab Resistance	Nuts/lb	% Kernel	Yield Per Plant (Kg)	Nut Dimension (mm)
1	Farley	AC	TE	Protogynous	Mid	Excellent	39	47	12	26
2	Schley	AC	TE	Protogynous	Mid	Excellent	49	48	9	24
3	Cape Fear	AC	AG	Protandrous	Early	Good	55	55	11	27
4	Sanber	AC	AG	Protogynous	Mid	Excellent	37	55	14	20
5	Wichita	AC	AG	Protogynous	Mid	Excellent	45	64	9	21
6	Kanza	AC	AG	Protogynous	Mid	Excellent	49	58	12	20
7	Cheyenne	AC	AG	Protandrous	Early	Excellent	61	47	16	24
8	Elliot	AC	AG	Protogynous	Mid-late	Excellent	71	53	11	22
9	Mohawk	AC	AG	Protogynous	Early	Good	62	48	16	21
10	Western	AC	AG	Protogynous	Early	Good	56	44	18	23
11	Mahan	AC	TE	Protogynous	Mid	Good	51	57	13	25
12	Shoshoni	AC	TE	Protogynous	Early	Good	56	54	13	24
13	Starking Hardy Giant	AC	UN	Protandrous	Mid	Good	61	53	9	22
14	Pawnee	AC	UN	Protandrous	Early	Excellent	54	57	16	24
15	Zhongshan 25	DC	SS	Protogynous	Early	Good	53	51	12	22
16	Zhongshan 39	DC	NB	Protogynous	Mid	Good	48	53	11	23
17	Zhongshan 40	DC	NB	Protandrous	Early	Excellent	49	49	10	21
18	Nanjing 148	DC	NB	Protogynous	Early	Excellent	57	52	11	24
19	Gan2	DC	NC	Protogynous	Early	Excellent	66	61	8	25
20	Gan3	DC	NC	Protogynous	Mid	Excellent	58	58	9	23
21	Gan4	DC	NC	Protogynous	Early	Excellent	57	52	6	21
22	Gan5	DC	NC	Protandrous	Early	Excellent	49	54	9	21
23	Gan6	DC	NC	Protogynous	Early	Good	51	57	7	22
24	Gan8	DC	NC	Protandrous	Mid	Good	53	55	6	19
25	Jinhua 1	DC	DL	Protogynous	Early	Excellent	55	56	9	22
26	Huangshan 1	DC	HS	Protogynous	Mid	Excellent	43	51	6	23
27	IW1	FP	NB	Protandrous	−	Excellent	61	52	20	22
28–67	IW2, IIE1, IIE4, IIE6, IIE7, IIE8, IIE9, IIE17, IIE18, IIE20, IIW1, IIW2, IIW11, IIW13, IIW13Lv, IIW21, IIIN3, IIIN6, IIIN12, IIIN19, IIIN55, IIIE7, IVE15, IVE16, IVE17, IVW28, IVW29, VE5, VE12, VW5, VI4, N704, W21, Shuita 2, Caoping, Lannan1, S401, Changfu, Changfulv, Mang 1	FP	NB	Protogynous	−	−	−	−	−	−
68–73	L1, L2, L3, L4, L5, LW1	FP	SY	Protogynous	−	−	49	−	−	−
74	LE15	FP	SY	Protandrous	−	Excellent	36	56	29	22
75	IIE32	FP	NC	Protogynous	−	Excellent	50	52	27	25
76	*Carya cathayensis*	OH	JD	Good overlap	Late	Good	34	35	−	−
77	*Juglans nigra*	OH	AG	Good overlap	Late	Good	33	41	−	−

^a^ Type: AC, cultivars introduced from America; DC, domestic seedling breeding cultivars, *i.e.*, Chinese seedling breeding cultivars; FP, fine pecan plants with good characters and no cultivar data; OH, other hickory accessions. ^b^ Origin: TE-Texas Experimental Station for Pecan, USA; AG-American Germplasm Gene Bank for Pecan; UN-University of Nebraska, USA; SS-Seedling selection in Nanjing, China; NB-Nanjing Botanical Garden Mem. Sun Yat-sen; NC-Nanchang, Jiangxi Province, China; DL-Dali, Yunnan Province, China; HS-The Forestry Science Institution of Huangshan; SY-Sun Yat-sen Mausoleum in Nanjing; JD-Jiande, Zhejiang Province, China.

### 2.1. ISSR and SSR Amplification

A screen of the 77 accessions using 10 selected ISSR primers (Table 2) yielded a total of 94 amplified bands in the range of 140–1950 bp; all of the bands were polymorphic (100%). The number of polymorphic bands per primer ranged from 7 (ISSR-22 and ISSR-56) to 12 (ISSR-35), with an average of 9.4 produced by each selected primer. The amplified ISSR fragments discriminated all 77 of the accessions. In this study, 14 bands were unique to a single genotype. For instance, the 1700-bp-long band amplified by the ISSR-5 primer was observed only in S69 (“L1”) (“S” indicates “sample”), and the 180-bp band amplified by the ISSR-64 primer and the 500-bp band amplified by the ISSR-65 primer were only observed in S12 (“Shoshoni”). Moreover, the 1,600-, 1,400-, and 700-bp bands amplified by the ISSR-35 primer, the 180-bp band amplified by the ISSR-45 primer, the 600-bp band amplified by the ISSR-56 primer, the 1,400-, 500-, 460-, and 380-bp bands amplified by the ISSR-61 primer and the 1,270-bp band amplified by the ISSR-62 primer were observed only in S77 (“*Juglans nigra*”).

**Table 2:** Selected ISSR markers tested in this study.

Primer name	Primer sequence (5′-3′)	Total bands	Ratio of polymorphic bands	Fragment size range (kb)
ISSR-5	(AC)_8_TG	10	10/10 (100%)	0.36–1.95
ISSR-22	(AC)_8_AA	7	7/7 (100%)	0.34–1.89
ISSR-26	(AC)_8_CC	9	9/9 (100%)	0.36–1.38
ISSR-35	(AG)_8_TA	12	12/12 (100%)	0.15–1.64
ISSR-45	(AC)_8_GC	11	11/11 (100%)	0.18–1.53
ISSR-56	(AG)_8_TT	7	7/7 (100%)	0.14–1.28
ISSR-61	(AG)_8_GT	10	10/10 (100%)	0.35–1.44
ISSR-62	(AG)_8_CA	9	9/9 (100%)	0.32–1.27
ISSR-64	(AG)_8_CG	10	10/10 (100%)	0.18–1.08
ISSR-65	(AG)_8_CC	9	9/9 (100%)	0.25–1.07
Total	−	94	94/94 (100%)	−
Mean	−	9.4	9.4/9.4 (100%)	−

Of the 19 SSR primers tested, eight producing clear and reproducible bands were chosen for further study (Table 3). A total of 70 fragments, ranging in size from 50 to 350 bp, were generated; all of them were polymorphic (100%) and approximated the expected size. The polymorphic bands produced by a single primer ranged from 2 (SSR-18) to 18 (SSR-7), with an average of 7. Similar to the ISSR analysis, there were no pair accessions that exhibited identical band patterns. Fourteen bands were unique to a single genotype. The unique 110-bp-long band amplified by the SSR-4 primer was identified in S35 (‘ⅡE17’), the 260-bp band amplified by the SSR-15 primer was only observed in S4 (“Sanber”) and the 150-, 140-, 130-, and 120-bp bands amplified by the SSR-16 primer were only produced in S76 (“*Carya cathayensis*”). In addition, the 80-bp band amplified by the SSR-4 primer, the 350-bp band amplified by the SSR-7 primer, the 210- and 160-bp bands amplified by the SSR-8 primer, the 260- and 200-bp bands amplified by the SSR-9 primer and the 110- and 100-bp bands amplified by the SSR-19 primer were only detected in S77 (“*Juglans nigra*”).

**Table 3:** Selected SSR markers tested in this study.

Primer name	GenBank accession	Primer sequence(forward primer 5′ to 3′, reverse primer 5′ to 3′)	Total bands	Ratio of polymorphicbands	Fragment size range (kb)	Annealing temp. (°C)
SSR-4	AY218217	AAATGGTGAGGAAGTGAAGATATGAA (forward)	9	9/9 (100%)	0.09–0.33	57
		GCCCCTTATACAGTTCTACCTCTCTC (reverse)				
SSR-7	AY218220	AATGAGATGACTACATACACAAGTCGG (forward)	18	18/18 (100%)	0.09–0.35	59
		GGGCTCGCATACCTTCATGA (reverse)				
SSR-8	AY218221	TGAACTCCAAAAGCCTCCTCTC (forward)	6	6/6 (100%)	0.11–0.23	56
		GTATTTGTATTTTTTCCTTGAGCTTTCTC (reverse)				
SSR-9	AY218222	AAAAGTTTTAGGGTTGTTTGCTCTCT (forward)	5	5/5 (100%)	0.14–0.26	56
		GTAAAGCCTACAACCTACAACAGTCTATG (reverse)				
SSR-15	AY218228	TGTAAATGCGTGCTATTGCTGAT (forward)	10	10/10 (100%)	0.09–0.28	54
		GAATAGACAAAGAAACGAAACTCATTGA (reverse)				
SSR-16	AY218229	TCTTCAGAAAAAACCCTTACCTCTCT (forward)	10	10/10 (100%)	0.06–0.16	56
		GAAAAATATAAACTCCCATAGTACCCACAT (reverse)				
SSR-18	AY218231	GGAGTTGTGGAAGCAGTGGA (forward)	2	2/2 (100%)	0.08–0.09	57
		GGACCATAAGAGTTTTGACCCTT (reverse)				
SSR-19	AY218232	CCCCAACTCAATTACAACCTCTTC (forward)	10	10/10 (100%)	0.05–0.11	55
		TGTTCATTCTGCACACACACAA (reverse)				
Total	−	−	70	70/70 (100%)	−	−
Mean	−	−	7	7/7 (100%)	−	−

### 2.2. Analysis of the Genetic Diversity of the Pecan Accessions

Based on the different origins of the germplasm, the 77 accessions were divided into four groups, as follows: the AC group comprising 14 cultivars introduced from America, the DC group comprising 12 Chinese seedling breeding cultivars, the FP group comprising 49 fine pecan plants with great characteristics and no cultivar data, and the OH group comprising two other hickory genotypes. With the purpose of detecting the extent of genetic diversity of the pecan accessions in this study more accurately, the four groups were organized into the following four clusters: Cluster I, which included only one group (AC); Cluster II, which included two groups (AC and DC); Cluster III, which included three groups (AC + DC + FP); and Cluster IV, which included all four of the groups (AC + DC + FP + OH). And with the purpose of discuss the genetic relatedness between ISSR and SSR, genetic parameters were analyzed using the ISSR and SSR markers separately and were listed in Table 4. The number of polymorphic loci and the number of alleles for both the ISSR and SSR markers gradually increased with an increase in the sample size. Results for both the ISSR and SSR markers revealed similar trends of genetic diversity among the four clusters, as follows: Cluster IV > Cluster III > Cluster II > Cluster I. Moreover, in this study, the ISSR and SSR markers showed almost the same efficiency, based on the results, indicating that the ISSR and SSR were all suitable for the genetic diversity analyses and identification of the pecan samples.

**Table 4:** Genetic diversity estimates of accessions by ISSR and SSR analyses.

Parameter	ISSR				SSR			
	Cluster Ⅰ	Cluster Ⅱ	Cluster Ⅲ	Cluster Ⅳ	Cluster Ⅰ	Cluster Ⅱ	Cluster Ⅲ	Cluster Ⅳ
*N*	69	79	82	94	59	61	64	76
*P* (%)	73.40	84.04	87.23	100	77.63	80.26	84.21	100
*A*	1.73 ± 0.44	1.84 ± 0.37	1.87 ± 0.34	2.00 ± 0.00	1.78 ± 0.42	1.80 ± 0.40	1.84 ± 0.37	2.00 ± 0.00
*Ae*	1.36 ± 0.36	1.42 ± 0.37	1.44 ± 0.36	1.46 ± 0.35	1.38 ± 0.33	1.38 ± 0.33	1.41 ± 0.33	1.42 ± 0.32
*H*	0.22 ± 0.19	0.25 ± 0.19	0.26 ± 0.18	0.27 ± 0.17	0.24 ± 0.17	0.24 ± 0.17	0.25 ± 0.17	0.26 ± 0.16
*I*	0.34 ± 0.26	0.38 ± 0.25	0.40 ± 0.25	0.42 ± 0.22	0.36 ± 0.25	0.36 ± 0.24	0.39 ± 0.24	0.41 ± 0.21
*Gst*	0.8286	0.9190	0.9729	0.9742	0.8113	0.8987	0.9678	0.9694
*Nm*	0.1035	0.0441	0.0139	0.0133	0.1163	0.0564	0.0166	0.0158

Cluster I, including only cultivars introduced from America; Cluster II, including cultivars introduced from America and Chinese seedling breeding cultivars; Cluster III, including all pecan cultivars and fine plants; Cluster IV, including all 77 accessions; *N*: number of polymorphic loci; *P*: percentage of polymorphic loci; *A*: observed number of alleles; *Ae*: effective number of alleles; *H*: Nei’s gene diversity; *I*: Shannon’s information index.

In Table 4, we can observe a large number of alleles. Pecan is diploid (2n = 2x = 32) with a genome size of 1.7pg/2 × DNA content (≈1,344 Mb). So this result may be caused by more than one sequence matched designed markers in chromosome and formed some kind of allele patterns, which can be observed in the experiment. The markers used in this article were developed by Grauke *et al.* in 2003 using a native pecan genotype “Halbert” and were evaluate for the genetic studies of 48 pecans and other hickory spp. [12]. Grauke’s experiment had showed the same result.

When analyzing the genetic diversity of all 77 of the accessions (Cluster IV), the ISSR results showed that the effective number of alleles (*Ae*) was 1.46, Nei’s gene diversity (*H*) was 0.27 and Shannon’s information index (*I*) was 0.42 [24]. The SSR results showed that the effective number of alleles (*Ae*) was 1.42, Nei’s gene diversity (*H*) was 0.26 and Shannon’s information index (*I*) was 0.41. These results indicated that a high genetic variance existed among the accessions. The ISSR and SSR markers both expressed a high level of polymorphism in distinction of the accessions. ISSRs were revealed as more efficient for pecan diversity evaluation compared with SSRs except with American introduced cultivars. The genetic variance analysis using the ISSR markers showed that the coefficient of gene differentiation (*Gst*) was 0.9742 and the gene flow (*Nm*) was 0.0133, whereas the genetic variance analysis using the SSR markers indicated that the coefficient of gene differentiation (*Gst*) was 0.9694 and the gene flow (*Nm*) was 0.0158. The coefficient of gene differentiation results showed that 97.42% and 96.94% of the differences existed among the cultivars and 2.58% and 3.06% of the differences existed within the cultivars, indicating that the pecan cultivars had significant diversity at the DNA level. The pecan is monoecious, that is, the male (catkin or staminate flower) and female (pistillate flower) flowers are borne separately at different locations on the same tree. However, within a pecan cultivar, pollen shedding usually does not closely overlap with the period when the stigma is receptive, which tends to ensure cross-fertilization rather than self-fertilization. Our results are consistent with this character of pecans and the results of previous studies [11,12,13,14,15]. Our results provide molecular data for the genetic improvement and breeding of pecan.

### 2.3. Dendrogram from ISSR, SSR and Combined Data

The UPGMA cluster analysis was firstly conducted using only the ISSR data and showed a two cluster structure: *Juglans nigra* and the other accessions. The other 76 accessions were divided into two groups: group I (containing 26 accessions) and group II (containing 50 accessions). The cultivars and fine plants were mixed together and scattered in two groups, but some patterns remained. The UPGMA cluster analysis based on the SSR data also revealing a structure of two clusters: *Juglans nigra* and the other accessions. The other 76 accessions were divided into three groups: group I (containing 73 accessions), group II (containing one accession) and group Ⅲ (containing two accessions).

We then combined the ISSR and SSR data because the combined data permit a more thorough analysis of the genetic variations [25]. The Nei genetic identities of the pecan cultivars are listed in Table 5. The accessions analyzed in this study represent a wide range of germplasms, cultivars introduced from America, main Chinese breeding cultivars and fine pecan plants. The genetic identity among the pecan cultivars ranged from 0.97 (between “Zhongshan 25” and “Huangshan 1”) to 0.59 (between “Gan 4” and “Gan 5”). *Juglans nigra* exhibited lower genetic identity than the pecan cultivars, ranging from 0.59 for “Mang 1” to 0.48 for “Mahan”. The average genetic identity among the American cultivars (0.79) was higher than the Chinese cultivars (0.75), indicating that the Chinese cultivars had a higher genetic diversity. Genetic variation may be caused by factors such as different locations, climates, and cultivation methods. The Chinese cultivars originated from the American cultivars, and, after introduction into China, the cultivars were affected by the local climate and cultivation methods. The most suitable cultivars were propagated and developed a great many characters. Thus, these cultivars have added abundant genetic diversity to the pecan germplasm. This result also highlights the role of the growing locality on the genetic diversity of pecan.

All 77 accessions in this study could be separated based on the ISSR and SSR fingerprints produced by one or more primers. *Juglans nigra* can be easily identified by many primers that it presented a lot of bands that all other accessions lacked. Other six accessions—*Carya cathayensis*, Sanber, Kanza, L1, IIW13Lv, and IVW28—could be identified through the presence of a single ISSR or SSR band that all other accessions lacked. All other accessions required at least two bands to be scored for an identification to be made. Therefore, ISSR and SSR markers have good potential for use in fingerprint pecan.

**Table 5:** Nei’s original measures of genetic identities of pecan cultivars.

1	Farley	-																								
2	Schley	0.83	-																							
3	Cape Fear	0.86	0.88	-																						
4	Sanber	0.79	0.88	0.82	-																					
5	Wichita	0.74	0.85	0.84	0.82	-																				
6	Kanza	0.81	0.90	0.85	0.84	0.82	-																			
7	Cheyenne	0.66	0.83	0.72	0.76	0.79	0.83	-																		
8	Elliot	0.83	0.88	0.85	0.80	0.79	0.89	0.76	-																	
9	Mohawk	0.66	0.82	0.76	0.78	0.80	0.79	0.75	0.72	-																
10	Western	0.84	0.84	0.85	0.78	0.76	0.80	0.69	0.86	0.71	-															
11	Mahan	0.81	0.86	0.83	0.79	0.79	0.90	0.78	0.88	0.75	0.82	-														
12	Shoshoni	0.89	0.83	0.84	0.75	0.77	0.79	0.65	0.84	0.68	0.85	0.81	-													
13	Starking H G	0.76	0.84	0.80	0.80	0.73	0.85	0.71	0.79	0.75	0.72	0.79	0.72	-												
14	Pawnee	0.71	0.83	0.80	0.82	0.83	0.81	0.76	0.75	0.76	0.75	0.75	0.74	0.72	-											
15	Zhongshan 25	0.94	0.82	0.85	0.80	0.73	0.81	0.64	0.81	0.66	0.82	0.78	0.86	0.77	0.68	-										
16	Zhongshan 39	0.81	0.82	0.81	0.80	0.70	0.79	0.64	0.79	0.65	0.79	0.78	0.78	0.73	0.69	0.83	-									
17	Zhongshan 40	0.79	0.80	0.81	0.81	0.78	0.84	0.78	0.78	0.73	0.76	0.81	0.75	0.75	0.78	0.78	0.78	-								
18	Nanjing 148	0.82	0.81	0.78	0.82	0.75	0.85	0.71	0.79	0.71	0.75	0.81	0.76	0.78	0.71	0.82	0.78	0.79	-							
19	Gan2	0.91	0.77	0.82	0.77	0.74	0.79	0.66	0.78	0.65	0.79	0.75	0.81	0.72	0.65	0.92	0.78	0.78	0.86	-						
20	Gan3	0.75	0.87	0.82	0.82	0.84	0.85	0.78	0.84	0.79	0.78	0.82	0.76	0.79	0.82	0.74	0.74	0.82	0.75	0.71	-					
21	Gan4	0.71	0.78	0.74	0.79	0.78	0.76	0.72	0.74	0.72	0.74	0.76	0.76	0.66	0.75	0.69	0.72	0.79	0.71	0.69	0.78	-				
22	Gan5	0.64	0.79	0.76	0.76	0.78	0.79	0.78	0.72	0.79	0.68	0.73	0.66	0.73	0.81	0.63	0.68	0.78	0.68	0.59	0.84	0.71	-			
23	Gan6	0.71	0.77	0.73	0.76	0.81	0.75	0.75	0.71	0.69	0.68	0.73	0.75	0.66	0.75	0.65	0.71	0.78	0.71	0.69	0.78	0.88	0.72	-		
24	Gan8	0.73	0.79	0.76	0.75	0.68	0.79	0.64	0.74	0.65	0.73	0.70	0.72	0.76	0.68	0.75	0.84	0.72	0.76	0.74	0.73	0.71	0.68	0.70	-	
25	Jinhua 1	0.66	0.76	0.72	0.79	0.71	0.77	0.67	0.68	0.70	0.61	0.66	0.64	0.70	0.72	0.68	0.72	0.72	0.71	0.67	0.76	0.72	0.77	0.76	0.76	-
26	Huangshan 1	0.94	0.81	0.86	0.79	0.75	0.82	0.64	0.82	0.67	0.83	0.79	0.85	0.76	0.69	0.97	0.81	0.80	0.84	0.95	0.75	0.69	0.64	0.66	0.74	0.65
Cultivar no.		1	2	3	4	5	6	7	8	9	10	11	12	13	14	15	16	17	18	19	20	21	22	23	24	25

**Figure 1:**
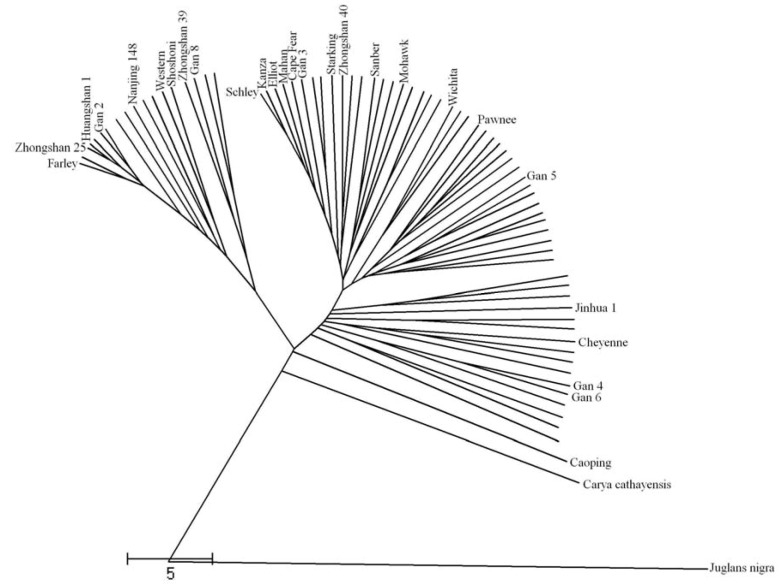
Dendrogram of 77 accessions derived from an UPGMA cluster analysis based on Nei’s distances using combined ISSR and SSR data.

The dendrograms constructed with ISSR, SSR or combined data were very similar, but with some differences which led to some better representation of the genetic relationship for cultivars and fine plants. The dendrogram constructed from both the ISSR and SSR similarity data showed a relatively indistinct grouping model among the different accessions (Figure 1). However, a few prominent groupings were discerned. The progeny were always grouped with the parents in all three of the dendrograms. “Schley”, “Mahan” and “Cape Fear” show a very close grouping relationship in all three of the dendrograms constructed from the ISSR, SSR and combined data. “Schley” was seedling selected from a nut planted circa 1881 by Delmas, at Scranton, Jackson County, MS, USA. This cultivar is used extensively in breeding and is the female parent of “Cape Fear”. “Mahan” is a well-known, older cultivar that originated from a nut plant by Chesnutt and has been widely used in pecan breeding [26,27]. Some research has proposed “Schley” as a parent of “Mahan” based upon the inheritance of unnamed characters. Our research also provided good support of this relationship for the high similarity between “Schley” and “Mahan” (0.86). “Mahan” is also the pollen parent of “Wichita” and “Mohawk”, whereas “Pawnee” originated from a controlled cross of “Mohawk” and “Starking H G”. Their group patterns were all in agreement with the pedigree relationships. Our results are also in accordance with the study of Grauke, L. who obtained a 0.63 (Jaccard’s coefficient) between “Mahan” and “Wichita” based on an SSR analysis [12]. “Kanza”, “Elliot” and “Schely” were always grouped together, suggesting their possible close genetic relationship. The progeny were all grouped with their parents, supporting the accuracy of the molecular markers.

A large number of Chinese pecan cultivars and fine plants are of unknown or questionable pedigree. This is because many were selected from seedling pecan plants that lacked a cultivar record and neither parent was known, or they were produced early in the century before efficient means of pollination control of this wind-pollinated species were established [27]. The applications of molecular marker techniques for pedigree determination in these cultivars and fine plants were very meaningful. We anticipate using the information gathered in this study to examine the pedigree origins of Chinese seedling breeding cultivars and fine plants. “Zhongshan 25”, “Huangshan 1”, “Gan 2” and “Nanjing 148” always grouped with “Farley”, showing their close relationship with “Farley” and also suggested their possible breeding source. “Gan 4” always grouped with “Gan 6”, indicating their close relationship. Using data obtained from this experiment may not fully identify the genetic pedigree of fine plants. But this experiment provided us a lot of useful data for understanding their genetic backgrounds. Some fine plants with great characters showed relatively lower similarity coefficients with other accessions. The fine plant, “Caoping”, was relatively far from all of the other pecan accessions in this study, showing its relatively far genetic distance with other pecan accessions, which may be due to its unique cultivation environment. This result was in accordance with the morphological characteristics of “Caoping”, such as an extremely high yield, a low alternate bearing tendency, smaller nuts and a good flavor. These fine plants have added good materials for breeding programs. From the result, we could see that most morphological traits in the dendrogram did not indicate a regular relationship with the clustering of the pecan accessions. Pecan had minor differences in morphological and economical traits except pollination, so identification of pecan cultivars by morphological characteriatics was very hard. This also indicated that molecular markers were very useful in pecan discrimination. However, we do discover an interesting phenomenon that economical traits such as the nut dimension sometimes had a relationship with morphological traits. Long nut dimensions were usually more popular than short nut dimensions in China. People like pecan nuts with a long-round shape. This kind of pecan nut usually could sell in higher price.

The dendrogram analysis reveals a definite gap between pecan and other hickories, as was expected on the basis of known levels of relatedness. *Carya cathayensis* shares the same genus with pecan (*Carya* Nutt. species) and has a relatively close relationship with the pecan accessions, whereas *Juglans nigra* is of a different genus (*Juglans* species), very far away from the other accessions.

## 3. Materials and Methods

### 3.1. Plant Materials

The cultivars used in the present study were obtained from America and China and had been cultivated in China for at least 2 years. The cultivars analyzed in this study were maintained in the scientific orchards of the Institute of Botany, Jiangsu Province and Chinese Academy of Sciences, Nanjing, China.

### 3.2. Total Genomic DNA Extraction and PCR

Total genomic DNA was extracted from young leaves collected in early spring using a DNAsecure Plant kit (TIANGEN Biotech Co., Ltd., Beijing, China). The quality of the total DNA was verified by gel electrophoresis (1% agarose gel) and quantified by spectrophotometry (Shimadzu, UV-2501PC, Japan). The DNA samples were stored at −20 °C. This genomic DNA was used for both the ISSR and SSR-PCR amplifications.

A total of 77 ISSR primers (Nanjing Sunshine Biotechnology Co., Ltd., Nanjing, China) were prescreened using two cultivars; 10 primers were selected based on polymorphism, quality and the reproducibility of the amplifications. The procedure for the ISSR marker amplification was modified from that of Zhao *et al.* The PCR was conducted in a 20 µL reaction volume containing 1× PCR buffer, 40 ng genomic DNA, 0.2 mM dNTP, 0.2 mM Mg^2+^, 0.4 mM primer and 1 U *Taq* DNA polymerase [27]. The PCR reactions were conducted in an Eppendorf AG thermal cycler (Germany). The amplification were conducted with an initial denaturation at 94 °C for 3 min, followed by 35 cycles of denaturation at 94 °C for 30 s, annealing at 54 or 56 °C for 30 s and an extension at 72 °C for 1 min; a final extension was conducted at 72 °C for 3 min. The amplification products were stored at 4 °C before analysis. The amplified products were separated on 1.5% (w/v) agarose gels containing approximately 0.5 µg·mL^−1^ ethidium bromide (EB) in 1× Tris-acetate-EDTA (1× TAE) buffer; the gels were then photographed under ultraviolet light (FR-200A, Shanghai Furi Science & Technology Co., Ltd., Shanghai, China).

The SSR assays were performed as described by Grauke *et al.* using eight primer pairs selected from among the 19 SSR primer pairs that were developed for pecan by Grauke *et al.* [12]. The prescreening was performed using three DNA samples; eight primer pairs showed good polymorphism and clear bands and were chosen for further analysis. The primers were synthesized by Shanghai Invitrogen Biotech, China. The amplification reactions were conducted in a 10 µL reaction volume containing 1× PCR buffer, 20 ng genomic DNA, 0.2 mM dNTP (plus Mg^2+^), 0.3 mM forward and reverse primers and 1 U *Taq* DNA polymerase. The SSR amplification were conducted with an initial denaturation at 94 °C for 3 min, followed by 35 cycles of denaturation at 94 °C for 30 s, annealing at the specific temperature (53 to 59 °C) shown in Table 3 for 30 s and extension at 72 °C for 1 min; a final extension was conducted at 72 °C for 3 min. The amplification products were stored at 4 °C before analysis. The amplified products were then separated by electrophoresis on 8% denaturing acrylamide gels using 1× TBE buffer at 230 V for 1.5 h or until the blue dye reached the buffer in the lower tank. The DNA bands were visualized by silver staining, as described by Bassam and Caetano-Anolles [28].

### 3.3. Data Analysis

Only distinct, well-resolved fragments were scored as present (1) or absent (0) for each of the ISSR and SSR markers in the 77 accessions. Two different matrices of the ISSR and SSR data were analyzed among the four clusters composed of different accessions using POPGENE Software version 1.31 [29]. The following genetic diversity parameters were estimated: the number of polymorphic loci (*N*), the percentage of polymorphic loci (*P*), the observed number of alleles (*A*), the effective number of alleles (*Ae*), Nei’s gene diversity index (*H*), Shannon’s information index of genetic diversity (*I*), the coefficient of gene differentiation (*Gst*), and the gene flow (*Nm*).

Clustering analysis was conducted using the unweighted pair group method with arithmetic average (UPGMA) and dendrograms were constructed for the ISSR, SSR and combined data, respectively, using the scorable fragments (The dendrograms of ISSR and SSR are showed in the Supplementary Data). In addition, a matrix of pecan cultivars was constructed using Nei’s original measures of genetic identities.

## 4. Conclusions

In summary, the genetic diversity analyses indicated that a high genetic variance existed among the 77 studied accessions. Pecan cultivars and fine plants had significant diversity at the DNA level. Perhaps due to the growing locality, Chinese cultivars had a higher genetic diversity than American introduced cultivars.

The dengrograms constructed with ISSR, SSR or combined data were very similar but showed very weak grouping association with morphological characters. However, the progeny were always grouped with the parents and in accordance with pervious research. Using data obtained from this experiment may not fully identify the genetic pedigree of fine plants, but provided us a lot of useful data for understanding their genetic backgrounds. Some interesting fine plants were identified for their unique genetic relationship with other accessions. The great diversity found among the Chinese cultivars and the interesting germplasm of fine pecan plants indicated that the Chinese cultivars and fine plants comprise variable sources of pecan genes for use in breeding programs. Thus, it is necessary to search among the Chinese pecan cultivars and fine plants with the goal of diversifying the available pecan gene pool.

All 77 accessions in this study could be separated based on the ISSR and SSR fingerprints produced by one or more primers. The results of our study also showed that ISSR and SSR techniques are both suitable for genetic diversity analyses and the identification of pecan resources.

To the best of our knowledge, we report here for the first time the genetic variation of Chinese-grown pecan cultivars and fine plants. The results reported here could have practical uses and may be useful for future studies.

## Supplementary Materials

Supplementary materials can be accessed on: http://www.mdpi.com/1420-3049/16/12/10078/s1.
