# Supplementary material for: Genetic Diversity and Identification of Chinese-Grown Pecan Using ISSR and SSR Markers
Source: Molecules. 2011 Dec 6;16(12):10078–92. doi: 10.3390/molecules161210078 (PMC6264290; doi:10.3390/molecules161210078)
Supplement: Supplementary file 1 [file molecules-16-10078-s001.doc]

**Supplementary table of Figure 1.**

| **No (from left to right)** | **Name** |
| --- | --- |
| 1 | **Farley** |
| 2 | **Zhongshan 25** |
| 3 | ⅤE5 |
| 4 | **Huangshan 1** |
| 5 | ⅢN19 |
| 6 | **Gan 2** |
| 7 | ⅡE8 |
| 8 | ⅡE17 |
| 9 | L2 |
| 10 | **Nanjing 148** |
| 11 | ⅠW1 |
| 12 | ⅡE6 |
| 13 | **Western** |
| 14 | **Shoshoni** |
| 15 | **Zhongshan 39** |
| 16 | **Gan 8** |
| 17 | ⅡE32 |
| 18 | L3 |
| 19 | **Schley** |
| 20 | **Kanza** |
| 21 | **Elliot** |
| 22 | **Mahan** |
| 23 | **Cape Fear** |
| 24 | **Gan 3** |
| 25 | ⅡW11 |
| 26 | ⅡW21 |
| 27 | **Starking H G** |
| 28 | **Zhongshan 40** |
| 29 | ⅣE16 |
| 30 | Ⅵ4 |
| 31 | **Sanber** |
| 32 | Changfulv |
| 33 | ⅡE18 |
| 34 | **Mohawk** |
| 35 | L4 |
| 36 | ⅠW2 |
| 37 | Lannan1 |
| 38 | ⅡW1 |
| 39 | **Wichita** |
| 40 | ⅡE4 |
| 41 | ⅡE1 |
| 42 | **Pawnee** |
| 43 | ⅤE12 |
| 44 | ⅡW2 |
| 45 | ⅡW13 |
| 46 | ⅢE7 |
| 47 | ⅣW28 |
| 48 | ⅡE7 |
| 49 | **Gan 5** |
| 50 | ⅣE17 |
| 51 | LW1 |
| 52 | ⅡW13Lv |
| 53 | ⅣW29 |
| 54 | Mang 1 |
| 55 | S401 |
| 56 | ⅢN55 |
| 57 | ⅣE15 |
| 58 | LE15 |
| 59 | ⅡE20 |
| 60 | ⅢN3 |
| 61 | ⅢN6 |
| 62 | **Jinhua 1** |
| 63 | ⅡE9 |
| 64 | L1 |
| 65 | **Cheyenne** |
| 66 | N704 |
| 67 | Shuita 2 |
| 68 | ⅤW5 |
| 69 | Gan 4 |
| 70 | Gan 6 |
| 71 | W21 |
| 72 | ⅢN12 |
| 73 | L5 |
| 74 | Changfu |
| 75 | Caoping |
| 76 | *Carya cathayensis* Sarg. |
| 77 | *Juglans nigra.* Linn. |

**Supplymentary Figure 1.** Dendrogram of 77 accessions derived from an UPGMA cluster analysis based on Nei’s distances using ISSR data.


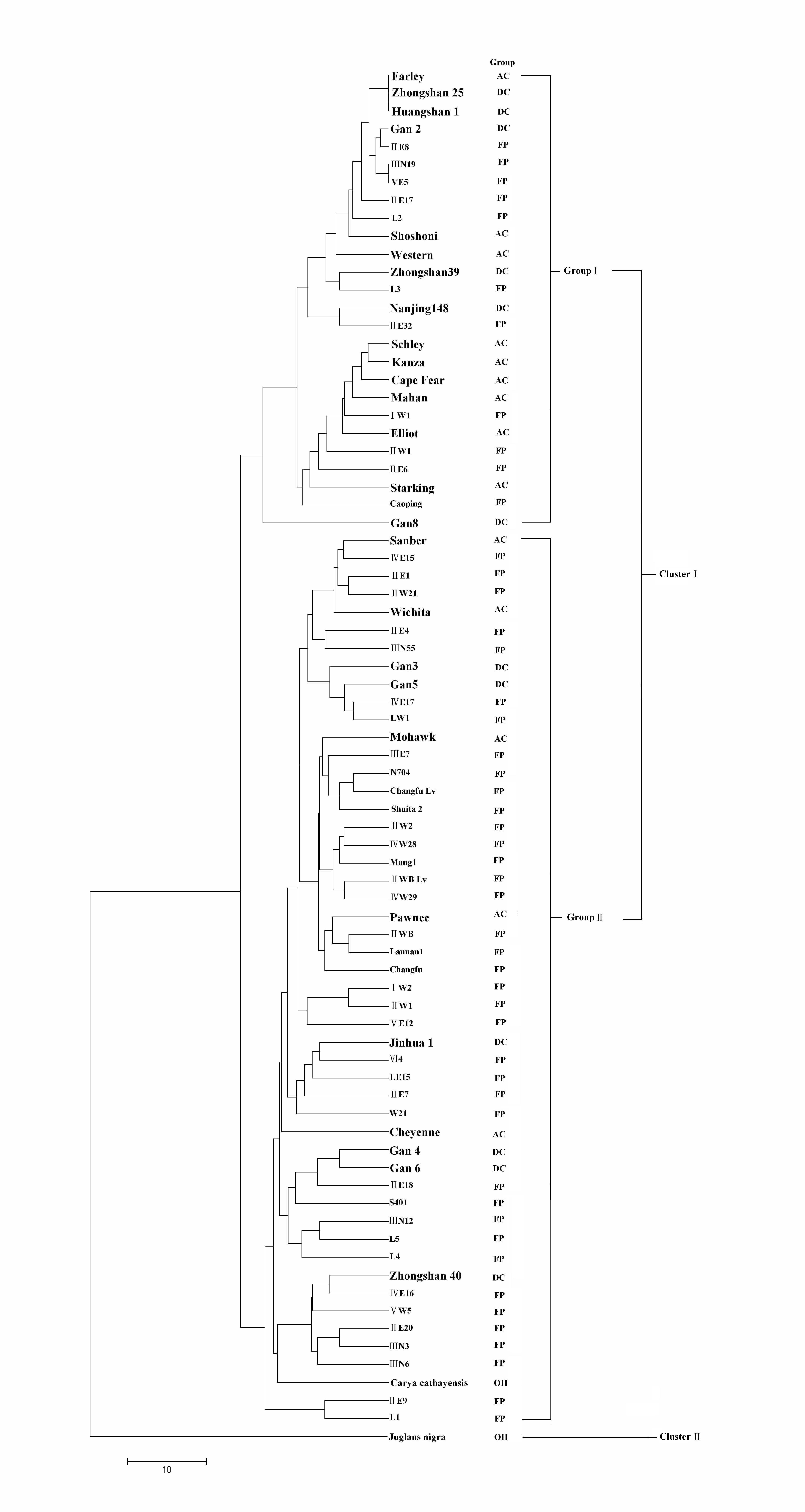


Group: AC, cultivars introduced from America; DC, domestic seedling breeding cultivars, *i.e.*, Chinese seedling breeding cultivars; FP, fine pecan plants with good characters and no cultivar data; OH, other hickory accessions.

**Supplymentary Figure 2.** Dendrogram of 77 accessions derived from an UPGMA cluster analysis based on Nei’s distances using SSR data.

**
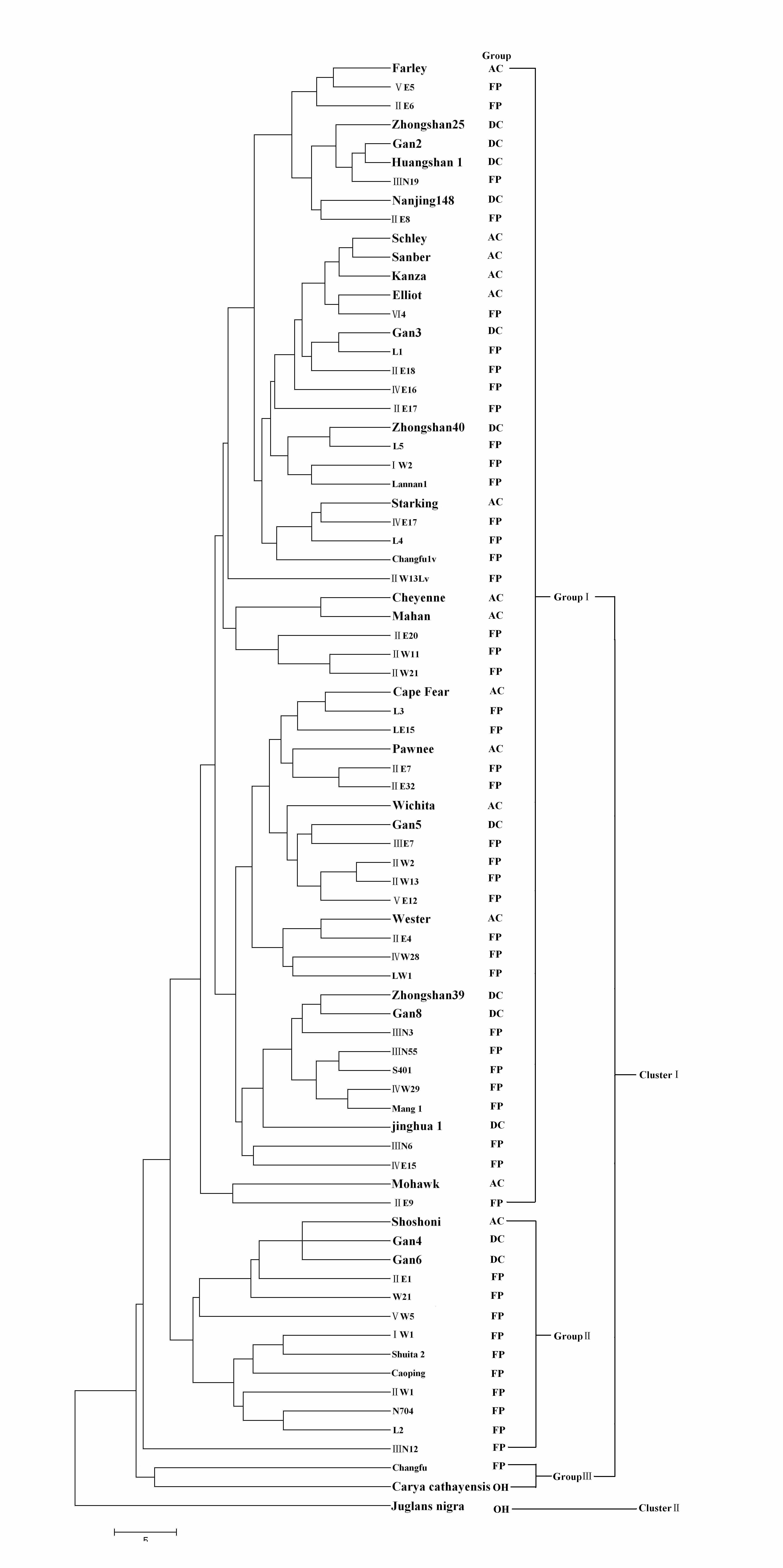
**

Group: AC, cultivars introduced from America; DC, domestic seedling breeding cultivars, *i.e.*, Chinese seedling breeding cultivars; FP, fine pecan plants with good characters and no cultivar data; OH, other hickory accessions.
